# Photo-polymerization as a new approach to fabricate the active layer of forward osmosis membrane

**DOI:** 10.1038/s41598-018-36346-8

**Published:** 2019-02-13

**Authors:** Pankaj M. Pardeshi, Alka A. Mungray

**Affiliations:** Chemical Engineering Department, Sardar Vallabhbhai National Institute of Technology, Ichchhanath, Surat, 395007 Gujarat India

## Abstract

A novel approach is employed to prepare the active layer of the forward osmosis membrane by the photo-polymerization method. The poly (ethylene glycol) phenyl ether acrylate (PPEA) and methacrylic acid (MAA) are used as monomers. The emphasis is given to analysing the effect of monomer concentration on chemical functional groups of active layer, thermal stability, surface morphology, roughness, interfacial free energy, organic fouling tendency and osmotic flux performance. The functional groups of the active layer are characterized by ATR-FTIR. Furthermore, thermal analysis (TGA/DTG) is performed to calculate grafting density and thermal stability of prepared FO membranes. Surface morphology and roughness are characterized by atomic force microscopy (AFM). Unlike control polyamide active layer membrane that suffered from organic fouling (28.14 ± 3.70% flux decline and 95% flux recovery), the photo-polymerized 75/25 active layer FO membrane demonstrated the low fouling propensity (2.77 ± 0.62% flux decline) and high flux recovery (nearly ~100%). The interfacial free energy and low fouling property of 75/25 FO membrane is also reflected in improved osmotic flux performance with 11.20 ± 0.79 L/g (AL-FS) and 8.41 ± 0.22 L/g (AL-DS) reverse solute flux selectivity (RSFS) (*J*_*w*_*/J*_*s*_) than control polyamide FO membrane (7.94 ± 0.22 L/g (AL-FS) and 7.64 ± 0.54 L/g (AL-DS)).

## Introduction

After proposing the idea and potential applications of forward osmosis (FO) by Sydney Loeb, FO has been seeing as an option for the water purification technology. This fascinating technology is being tried in many areas such as wastewater treatment, food processing, and desalination of seawater and brackish water. Some of them have also proved their applicability at commercial level. The attraction towards FO is understandable since it doesn’t required an external source of energy for the mass transfer and easy control on membrane fouling^1^.

An economic viability of every membrane process largely depends on the efficiency of a membrane. Despite having a number of researches on FO membrane preparation and modification, only the CTA FO membrane developed by Hydration technology Inc. (HTI) and polyamide thin film composite membrane (TFC) are commercially dominant in the FO membrane market^1^. This might be the only hurdle for FO to emerge as a new and effective water production technology.

Even now, attempts are being made to mitigate the identified disadvantages of the cellulose triacetate (CTA) FO membrane as if low water flux, low salt rejection, narrow pH range, and susceptibility to bio-fouling^2^, by FO scientists. Dabaghian *et al*.^3^ suggested a modification for a cellulosic membrane with carboxylic and amine functionalized carbon nanofiber (CNF), and a result in terms of water flux was increased by two-fold compared with a commercial CTA membrane. Wang *et al*.^4^ came up with the concept of free standing cellulose triacetate/graphene oxide (GO) composite membrane. Increased water flux and the anti-biofouling property were attributed to the absence of a support layer and incorporation of GO.

The thin film composite membrane (TFC) for FO was first developed in Elimelech’s lab^5^ by applying the layer of polyamide on the porous polysulfone substrate (PSf). However, a high rate of reverse salt flux (*J*_*s*_) made a TFC-FO membrane more vulnerable for selectivity than the TFC-RO membrane. Furthermore, the TFC-FO membrane has comparatively high fouling propensity, due to ridge-and-valley surface morphology, hydrophobicity, and a high density carboxylic group^6^. After realizing the importance of the polyamide active layer and disabilities of the TFC-FO membrane, many researchers accepted the need of surface modifications of TFC-FO membrane. Most of the researchers started with an approach like surface chemical modification and surface modification with nano-materials. Lu *et al*.^7^, modified the TFC-FO membrane chemically by Jeffamine and poly (ethylene glycol) derivative, and demonstrated the improved fouling resistance and water flux. Another recent study by Hegab *et al*.^8^, proposed surface chemical modification by attaching graphene oxide (GO) nano-sheets onto the surface of the TFC-FO membranes using the bio-adhesive polydopamine (pDA). The graphene oxide (GO) nano-sheets reduced and immobilized onto the membrane surface by self-assembly and oxidative polymerization of pDA. This modification resulted in an increased water flux, reverse solute flux selectivity and anti-biofouling properties. The growing curiosity in the nanotechnology and its imaginable application in many fields triggered the FO scientists to use nanoparticles for surface modification of the TFC-FO membrane. Tiraferri *et al*.^9^, altered the surface properties of the TFC-FO membrane by binding superhydrophilic silica nanoparticles. Soroush *et al*.^10^, tried to modify the surface of TFC FO membrane by silver nanoparticle (AgNPs)-decorated graphene oxide (GO) nanosheets. This proposed approach contributed in a rising hydrophilicity and antibacterial properties of the membrane. Liu and Hu^11^, have also contributed by modifying the surface of TFC-FO membrane using Mussel-inspired dopamine chemistry and silver nanoparticles (Ag NPs) to mitigate the effect of biofouling.

The number of publications on the surface modification of a commercial FO membrane is continuously growing exponentially. Regardless, all the proposed modifications for the commercial FO membranes are still limited to the lab scale. Consequently, there is a need to explore the other FO membrane fabrication methods.

The photo-polymerization technique has adequate potentials to synthesize high performance active layer of membranes. There are some specific advantages of the photo-polymerization technique such as: (i) it is possible to achieve high selectivity of photo-polymerization reaction under insignificant conditions (at low or ambient temperature), (ii) it is possible to perform a photo-polymerization reaction using no catalyst or a solvent, (iii) it is appropriate to small and large scale preparation processes, and (iv) it is low energy, non-polluting and a low cost process.

This method has been successfully used to fabricate as well as to improve the performance of polymeric membranes in gas separation, pervaporation, ultra- and microfiltration processes^12,13^. To the best of author’s knowledge, no study has been found on the use of the photo-polymerization method for the preparation of the active layer of FO membrane.

The main objective of the current study is to synthesize novel active layer FO membrane using the photo-polymerization technique. The monomers, poly (ethylene glycol) phenyl ether acrylate (PPEA) and methacrylic acid (MAA) are used with a ratio of (PPEA/MAA), 100/0, 75/25, 50/50, 25/75 and 0/100 to prepare active layer on optimized support membrane from our previous work^14^. The prepared FO membranes are characterized by ATR-FTIR for the surface functional groups, TGA/DTG for the thermal stability and grafting density, AFM for the surface morphology and roughness, and contact angle for the hydrophilicity and interfacial free energy. Finally, the performance of the prepared FO membranes are analysed by interfacial free energy, organic fouling tendency and osmotic flux performance. Furthermore, the performance of the prepared FO membranes are compared with the control polyamide FO membrane i.e. polyamide active layer on an optimized support membrane from previous work^14^.

## Results and Discussion

### FTIR of photo-polymerization of PPEA and MAA

**Figure 1:**
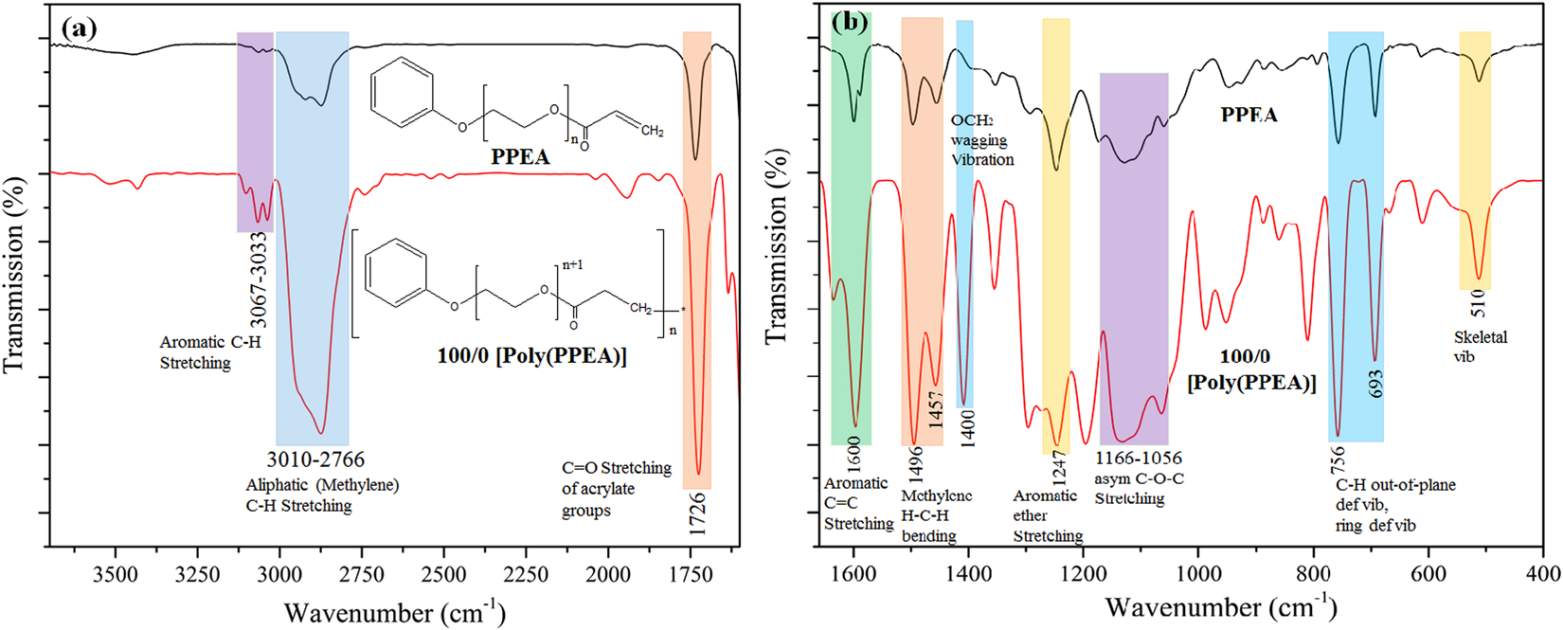
FTIR spectra of PPEA and Poly (PPEA) (100/0) (**a**) in 4000-1700 cm^−1^ wavenumber region and (**b**) in the fingerprint region.

**Figure 2:**
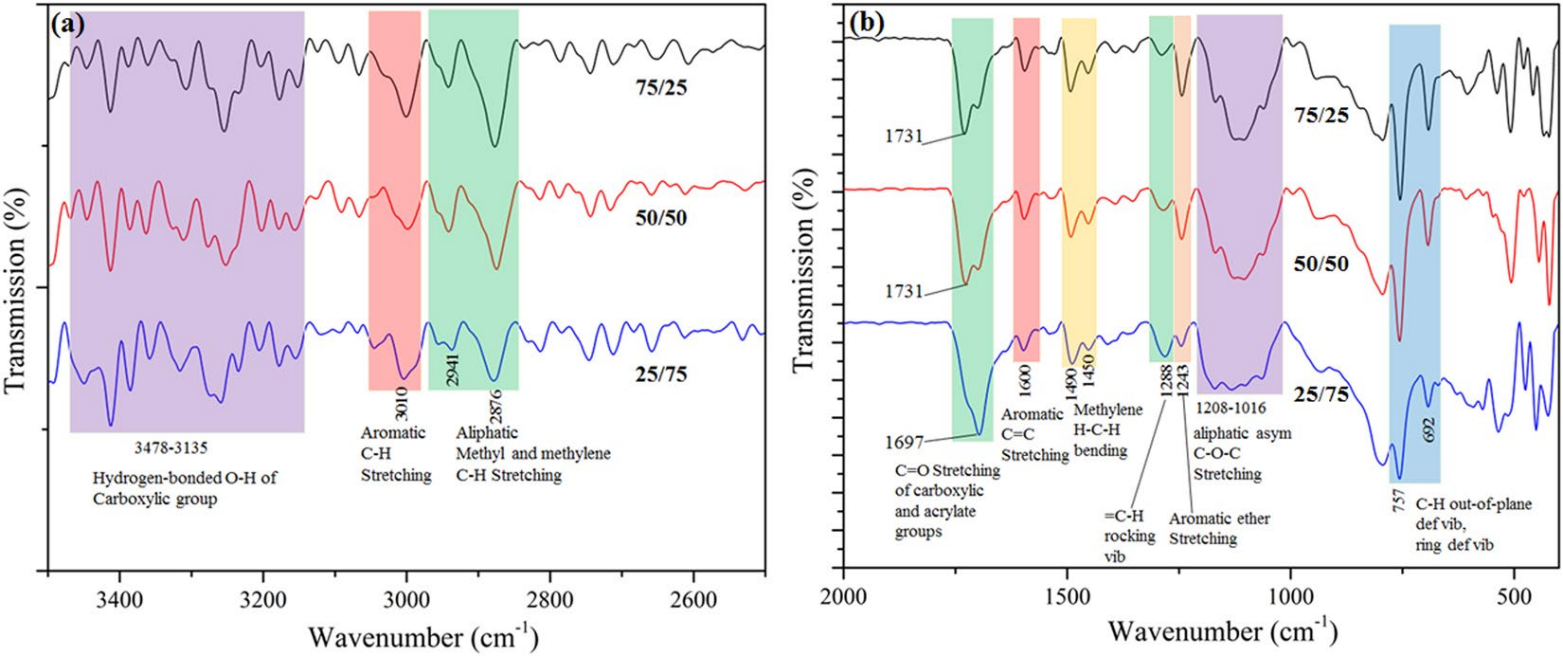
FTIR spectra of PPEA/MAA ratio of 75/25, 50/50 and 25/75 (**a**) in 4000-2500 cm^−1^ wavenumber region and (**b**) in the fingerprint region.

**Figure 3:**
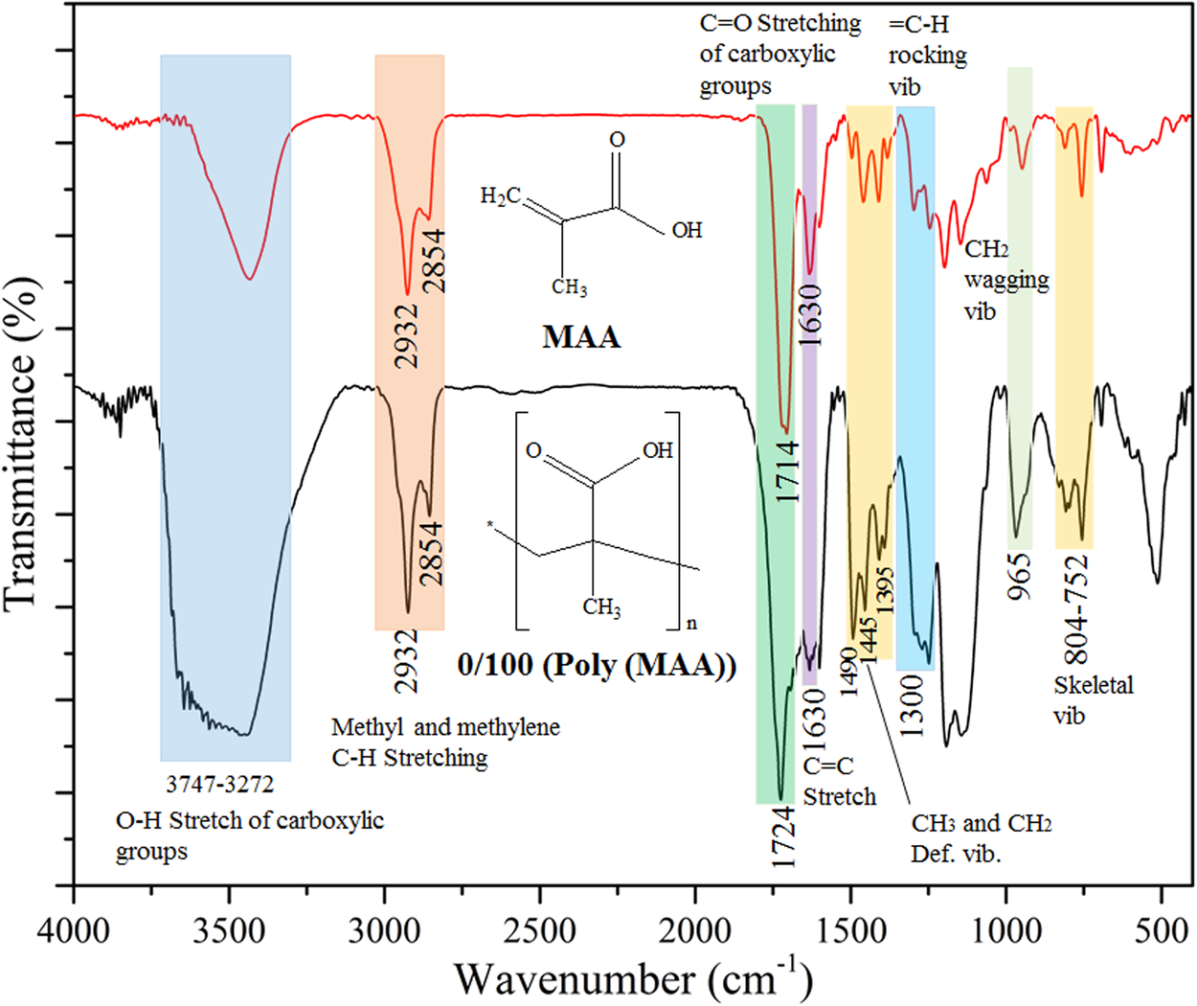
FTIR spectra of MAA and Poly (MAA) (0/100).

ATR-FTIR spectroscopy is used to analyse the photo-polymerization of PPEA and MAA. The FTIR spectra of PPEA monomer and Poly (PPEA) are presented in Fig. 1. The FTIR spectra of PPEA/MAA ratio of 75/25, 50/50 and 25/75 are shown in Fig. 2 and the FTIR spectra of MAA monomer and Poly (MAA) are given in Fig. 3.

As a comparison between the monomer (PPEA and MAA), and the polymer (Poly (PPEA) and Poly (MAA)), the only difference is observed in the transmittance of each wavelength areas. In Figs 1 and 3, Poly (PPEA) and Poly (MAA) have shown a lower transmittance than monomer PPEA and MAA. It is due to the fact that polymer has a long chain and resulted in a more IR absorption than the monomer.

In Fig. 1(a), the broad peak range at 3010–2766 cm^−1^ corresponds to aliphatic (methylene) C-H stretching. The small intensity peak at 3067 cm^−1^ and 3033 cm^−1^ are assigned to aromatic C-H stretching. The sharp peak at 1726 cm^−1^ is an evidence of C=O of acrylate group. The further evidences of all the functional groups of PPEA are observed in the fingerprint region (Fig. 1(b)) of FTIR spectra. The sharp peak at 1600 cm^−1^ is an indication of the aromatic C=C stretching. The methylene H-C-H bending indicates at small peak 1496 and 1457 cm^−1^. All small peaks at 1400 cm^−1^, 1247 cm^−1^, 1166–1056 cm^−1^, 756 cm^−1^, 693 cm^−1^ and 510 cm^−1^ are allotted to OCH_2_ wagging vibration, aromatic ether stretching, asymmetric C-O-C stretching, C-H out of plane deformation vibration or ring deformation vibration and skeletal vibration^15^. The FTIR spectral information of photo-polymerization of PPEA can be used to propose a plausible mechanism of photo-polymerization of PPEA as shown in Fig. S1 (Supporting Information).

Figure 2(a,b) shows the FTIR spectra of the mixture PPEA/MAA in the ratios 75/25, 50/50 and 25/75. In the spectral range 3478–3135 cm^−1^ (Fig. 2 (a)), the bonded O-H of a carboxylic group of MAA has shown a distinct number of peaks instead of a broad one peak of O-H. This might be due to the branched structure of produced poly (PPEA-co-MAA). The aromatic C-H stretching at 3010 cm^−1^, and aliphatic methyl and methylene C-H stretching at 2941 and 2876 cm^−1^ is confirmed the polymerization of PPEA and MAA. The invariant C=O functionality at 1731–1697 cm^−1^ is evidence of carboxylic and acrylate group of PPEA and MAA. In fingerprint region (Fig. 2(b)), the reduced intensity of C=C stretching peak at 1600 cm^−1^ is related to the aromatic ring of PPEA. Another invariant peak of methylene H-C-H bending at 1490 and 1450 cm^−1^ are confirmed the polymerization of PPEA and MAA. The reduced intensity of aromatic ether stretching at 1243 cm^−1^ and aromatic ring deformation at 757 and 692 cm^−1^ confirmed the decreased PPEA monomer concentration. The information from FTIR spectra of 75/25, 50/50 and 25/75 is used to predict the formation mechanism of PPEA-co-MAA polymer as shown in Fig. S2 (Supporting information).

In Fig. 3, the broad range of spectra at the peak 3747–3272 cm^−1^ presented the bonded O-H stretching vibration typical of the carboxylic group. The peaks at 2932 cm^−1^ and 2854 cm^−1^ are evidence of methyl and methylene C-H stretching. The C=O stretching vibration is typical of the acid carbonyl groups assigned at 1724 cm^−1^ and 1714 cm^−1^. The CH_3_, CH_2_ deformation vibrations spectra are clearly detected at 1490, 1445 and 1395 cm^−1^. The region at 1300, 965 and 804–752 cm^−1^ are assigned to the =C-H rocking vibration, CH_2_ wagging vibration and skeletal vibration. Similarly, On the basis of FTIR spectra of MAA and Poly (MAA) (0/100), the plausible mechanism of photo-polymerization of MAA is predicted as given in Fig. S3 (Supporting information).

### TGA/DTG analysis and grafting density of prepared active layer polymer

**Figure 4:**
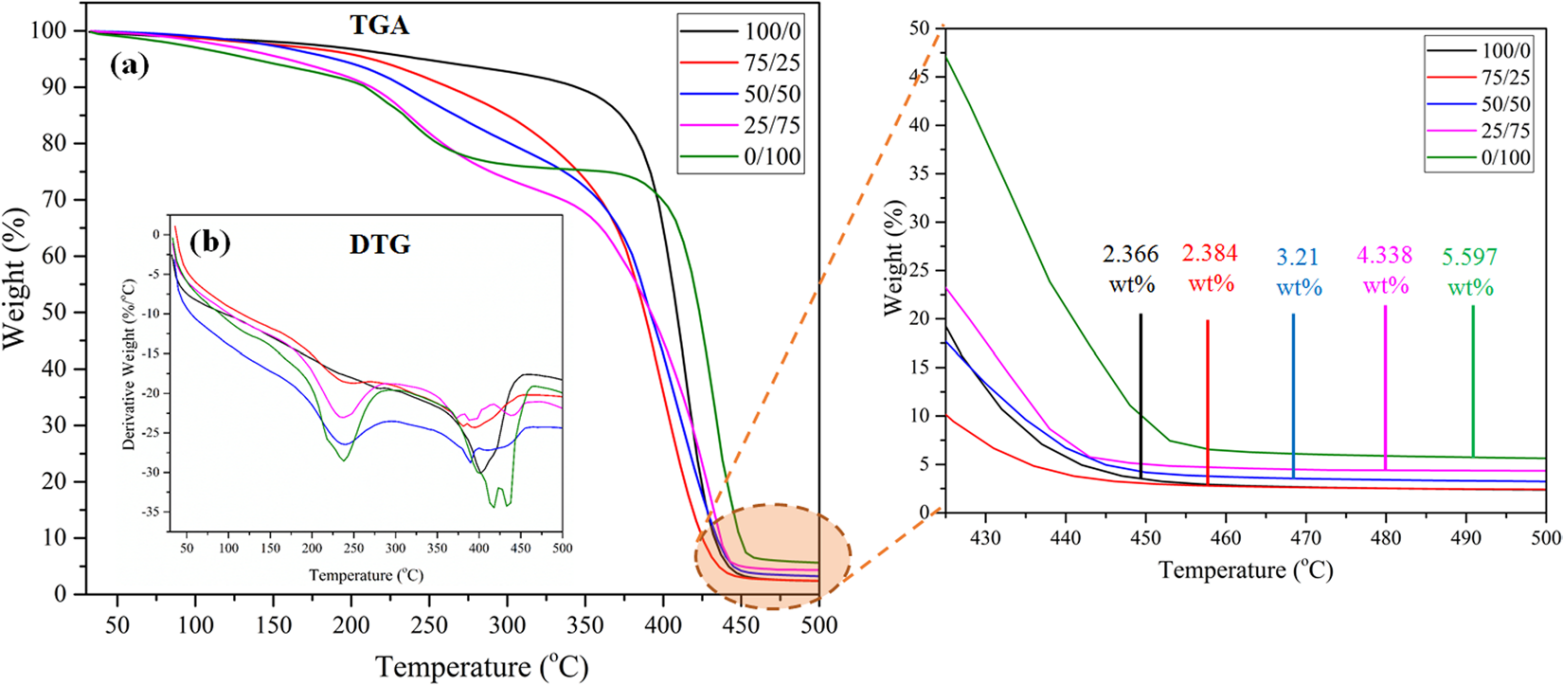
(**a**) TGA thermograms (**b**) inset DTG and (**c**) enlarged portion of TGA of 100/0, 75/25, 50/50, 25/75 and 0/100 active layer.

Figure 4 shows the TGA/DTG analysis of the prepared active layer to evaluate the thermal stability and grafting density with the different concentration ratios of PPEA and MAA. In TGA analysis, the sample is heated at a constant rate and the weight changes are measured (decomposition of material) while DTG thermograms measured the rate of mass loss.

In the case of 100/0 (Poly (PPEA)), after evaporation of physically adsorbed moisture and residual unreacted monomer (from ~32 °C to 187 °C, corresponding to 1.88 wt% loss), only one major degradation step is occurred from ~300 °C to ~450 °C (Fig. 4(a)). The most of the PPEA molecule occupied by the polyethylene glycol (PEG) chain and PEG has a simple linear chain bond structure which contains -C-O- (66%) and -C-C- (33%) in the backbone chain^16^. Therefore, the thermal degradation of 100/0 (Poly (PPEA)) is mainly occurred due to the degradation of the PEG chain.

In contrast, the 0/100 (Poly (MAA)) decomposed in two major step (Fig. 4(a)), the first step is started at ~200 °C due to depolymerization of Poly (MAA) resulted in a small yield of monomer. Second major step is initiated at ~370 °C due to an elimination of water molecule between a pair of carboxylic group^17^.

As the concentration ratio of PPEA/MAA changes from 0/100 to 100/0, the increasing concentration of PPEA in PPEA-co-MAA polymer backbone is resulted in the increased thermal stability. The 100/0 (poly (PPEA)) almost burnt out below 500 °C leaving behind only 2.366 wt% residue (Fig. 4(c)). The increased concentration of MAA resulted in an enhanced residue wt% as shown in Fig. 4(c). Consequently, the weight loss from the 75/25, 50/50 and 25/75 at 500 °C is used to approximate the amount of PPEA covalently attached to the MAA^18^.

Calculation for 75/25, {wt% of 0/100 (Poly (MAA)) − wt% of 75/25 (Poly (PPEA-co-MAA))} at 500 °C, 5.597 − 2.384 = 3.21 wt% of PPEA. Based on 1 g of 75/25 sample, the percentage of PPEA is 57.35% and percentage of MAA is 42.64%.

$$\begin{array}{rcl}Moles\,of\,PPEA & = & 1g\,of\frac{75}{25}\,sample\times \frac{0.5735\,g\,PPEA}{1\,g\,of\frac{75}{25}\,sample\,}\times \frac{1\,mole\,PPEA}{324\,g\,PPEA}\\  & = & 1.77\times {10}^{-3}\,mole\,of\,PPEA\\ Moles\,of\,MAA & = & 1g\,of\frac{75}{25}sample\times \frac{0.4264\,g\,MAA}{1\,g\,of\frac{75}{25}\,sample}\times \frac{1\,mole\,MAA}{86.06\,g\,MAA}\\  & = & 4.95\times {10}^{-3}\,mole\,of\,MAA\\ Grafting\,Density & = & \frac{1.77\times {10}^{-3}\,mole\,of\,PPEA}{4.95\times {10}^{-3}\,mole\,of\,MAA}\\  & = & 0.357\,mole\,PPEA\,per\,mole\,of\,MAA\\  & = & 357\,PPEA\,molecule\,per\,1000\,molecule\,of\,MAA\end{array}$$

The calculated grafting density of 75/25 polymer was 357 PPEA molecule per 1000 molecule of MAA.

Similarly, the calculated grafting density of 50/50 and 25/75 polymer are 197.6 PPEA molecule per 1000 molecule of MAA and 77.11 PPEA molecule per 1000 molecule of MAA respectively.

It has been profoundly studied that the covalent bonding between monomers plays a critical role in the development of structural and mechanical properties^18^ and these properties may be responsible for the change in surface properties of a membrane. These quantitative data of grafting density (covalent attachment of PPEA molecule to the MAA molecule) can be important to elucidate optimum concentration and the molecular weight of monomers to define structural and mechanical properties of the active layer.

### Atomic force microscopic

The AFM is preferred for surface morphological characterization of all the membranes instead of scanning electron microscopy (SEM). As a matter of fact, for the SEM analysis membrane samples need to go through an extensive sample preparation process that can damage the surface structure of the membrane. Furthermore, SEM is less appropriate for a very thin active layer because it is mostly overcome by the images of substrate background^19^.

The surface of polyamide membrane appeared as typical hills and valley structure and it has extensively studied by AFM analysis^9,20^.

**Figure 5:**
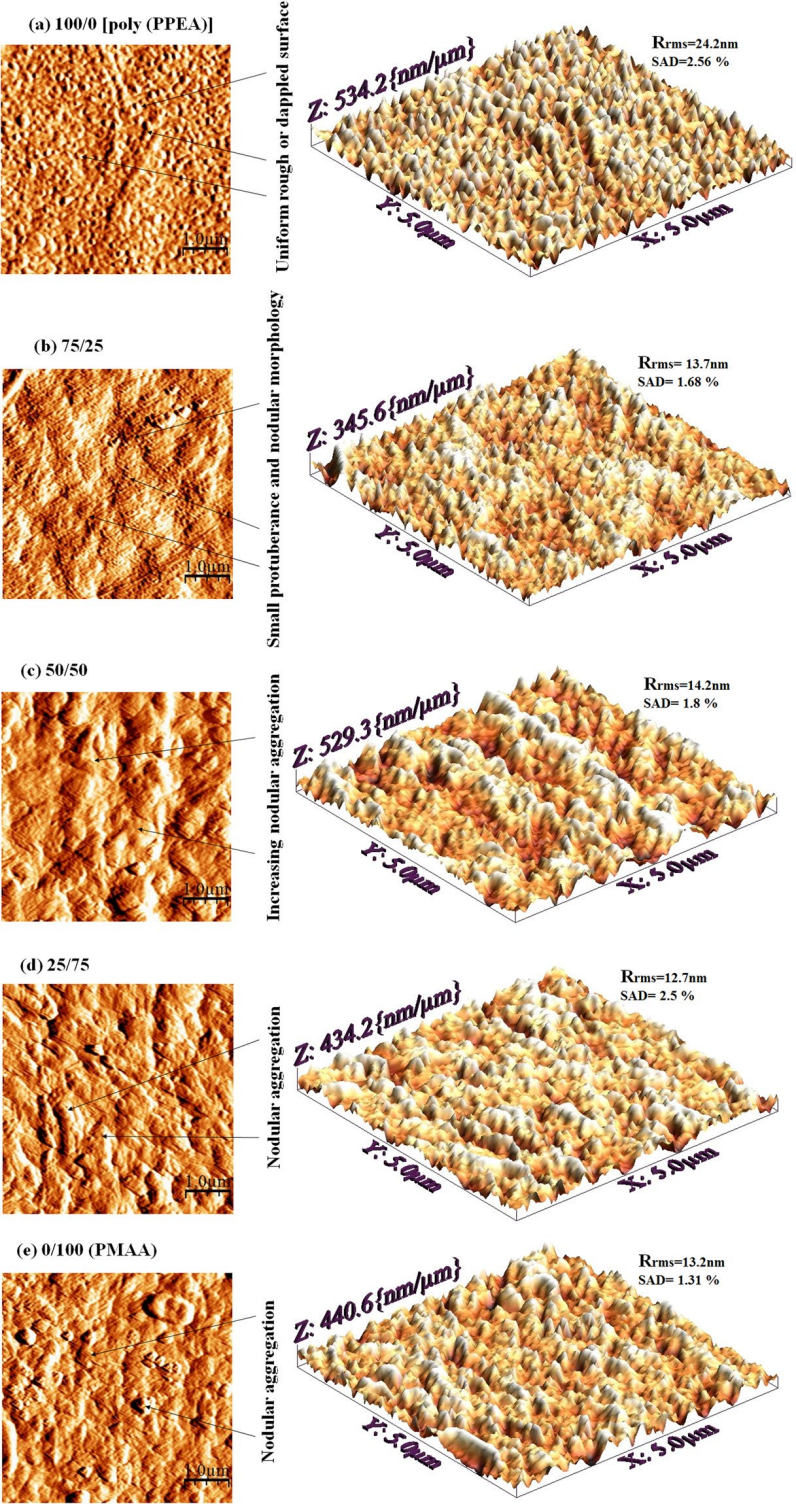
Surface morphology of active layer prepared by different PPEA/MAA ratio observed with AFM (**a**) 100/0 (**b**) 75/25 (**c**) 50/50 (**d**) 25/75, and (**e**) 0/100.

Figure 5 shows two dimensional and three dimensional surface morphology images of 5 µm scan for a different concentration of PPEA/MAA active layer. In a comparison of the polyamide surface images of TFC-FO membrane^9,20,21^, the distinct surface morphology is observed for PPEA/MAA active layer membrane. As shown in Fig. 5(a), the homogeneous surface morphology containing a rough or dappled surface is displayed for the 100/0 ratio of PPEA/MAA active layer. It may be due to the uniform photo-polymerization which result in an utmost degree of grafting and adequate coverage of PPEA on the surface of the substrate. As the ratio of PPEA/MAA varies or concentration of PPEA decreases and MAA increases, the appearance of the protuberance and nodular features on the surface of the membrane is observed. A small enhancement in the concentration of MAA as shown in Fig. 5(b), cause alteration of surface morphology from the dappled to a small nodular structure. It has also been observed that as the concentration of MAA increases, the nodular aggregation is enhanced as shown in Fig. 5(c–e). The nodular structure formation mechanism is critically explained by Wienk *et al*.^22^ on the surface of the ultrafiltration membrane as a function of polymer precipitation rate. This mechanism may apply to explain the nodular formation as the concentration of MAA increased in PPEA/MAA ratio. The photo-polymerization of the homogeneous mixture of monomer (PPEA and MAA), CQ as photo-initiator and EDMAB as an accelerator result into precipitation of inter-polymer complex (PPEA-co-MAA polymer). Whereas, the rate of precipitation of inter-polymer complex might have substantially affected by the concentration of MAA and hence aggregated nodular morphology is formed.

The AFM is also utilized to compare the roughness of all PPEA/MAA membranes. Comparatively uniform surface roughness with root-mean-square (RMS) roughness (R_rms_) of 24.2 nm and surface area difference (SAD) 2.56% is observed for 100/0 membrane. Increasing concentration of MAA leads to a relatively smoother surface i.e. 13.7, 14.2, 12.7, 13.2 nm R_rms_ and 1.68, 1.8, 2.5, 1.31% SAD for 75/25, 50/50, 25/75, 0/100 respectively lower than 100/0 as shown in Fig. 5(b–e). It has already been recognized that the rough surface of the membrane is more susceptible to accumulate the colloidal particles than a smooth surface. Therefore, it can be expected that 75/25, 50/50, 25/75 and 0/100 membrane will show good antifouling properties than 100/0 membrane.

It is important to mention that the dry sample is used to perform AFM analysis, and these roughness values may change in hydration condition. Nevertheless, the decreasing roughness as a concentration of MAA increased is not anticipated, and further study on the effect of different conditions on morphology or roughness changes needs to be carry forward to explore the causes of morphology changes.

### Interfacial free energy study

The foulant adhesion is a noticeable reason for membrane fouling. Currently, the membrane surface hydrophilicity/hydrophobicity is the primary parameter to substantiate the fouling control strategies. It has always been mentioned that more hydrophilic membrane is less prone to the surface fouling. In many previous membrane studies, the surface hydrophilicity of membranes has been considered equivalent to the water contact angle. Even so, the information from the water contact angle method alone is not capable to explain membrane fouling behavior^23,24^. The causes of foulant adhesion on the membrane surface have been well elucidated by a complex quantitative relationship of forces including the Lifshitz–van der Waals forces (LW), electrostatic double layer (EL) interactions and acid–base (AB)^23–25^. It has been reported and suggested that an acid-base (AB) interaction between membrane surface and foulant plays a dominant role, and fouling mitigation strategies should be concerned with the improvement of membrane surface ${\gamma }_{M}^{-}\,\,$and ${\rm{\Delta }}{G}_{MLM}^{TOT}$instead of only water contact angle^26^.

The interfacial free energy of interaction with water ${\rm{\Delta }}{G}_{MLM}^{TOT}$ is summarized in Table 1 for each membrane. A quantitative measure of the surface wettability is indicated by the sign and magnitude of ${\rm{\Delta }}{G}_{MLM}^{TOT}$. A positive value of ${\rm{\Delta }}{G}_{MLM}^{TOT}$shows a hydrophilic membrane while a negative value designates a hydrophobic membrane.

**Table 1:** The surface tension parameters (mJ/m^2^) for prepared FO membranes.

Membrane	${{\boldsymbol{\theta }}}_{{\boldsymbol{water}}}$ (°)	${{\boldsymbol{\theta }}}_{{\boldsymbol{gly}}}$ (°)	${{\boldsymbol{\theta }}}_{{\boldsymbol{diiod}}}$ (°)	*r* = (1 + SAD)	${{\boldsymbol{\gamma }}}_{{\boldsymbol{M}}}^{{\boldsymbol{LW}}}$ mJ/m^2^	${{\boldsymbol{\gamma }}}_{{\boldsymbol{M}}}^{+}$ mJ/m^2^	${{\boldsymbol{\gamma }}}_{{\boldsymbol{M}}}^{-}$ mJ/m^2^	${{\boldsymbol{\gamma }}}_{{\boldsymbol{M}}}^{{\boldsymbol{AB}}}$ mJ/m^2^	${{\boldsymbol{\gamma }}}_{{\boldsymbol{M}}}^{{\boldsymbol{TOT}}}$ mJ/m^2^	${\rm{\Delta }}{{\boldsymbol{G}}}_{{\boldsymbol{MLM}}}^{{\boldsymbol{TOT}}}$ mJ/m^2^
Control Polyamide	110 ± 2.5	83.5 ± 1.6	30.25 ± 1.4	1.23^9^	36.84	0.0045	0.222	0.063	36.90	−95.18
100/0	24.61 ± 1.2	53.72 ± 2.4	23.02 ± 1.2	1.0256	45.72	0.390	63.54	9.95	55.70	42.95
75/25	22.76 ± 2.5	45.31 ± 1.3	23.17 ± 1.7	1.0168	46.04	0.00185	56.44	0.64	46.70	40.37
50/50	36.77 ± 2.8	49.96 ± 3.4	30.54 ± 1.2	1.0180	43.28	0.005	45.28	0.95	44.20	26.15
25/75	68.78 ± 1.1	74.56 ± 1.0	34.80 ± 1.0	1.0250	41.20	0.70	21.67	7.80	49.00	−12.78
0/100	84.34 ± 3.0	105.13 ± 1.4	48.03 ± 1.2	1.0131	35.00	7.756	27.30	29.10	64.10	−1.52

The control polyamide FO membrane is found low surface energetic (${\gamma }_{M}^{TOT}$ = 36.90 mJ/m^2^) and hydrophobic (${\rm{\Delta }}{G}_{MLM}^{TOT}$ = −95.18 mJ/m^2^) even though having relatively wetting surface. The surface properties of prepared FO membrane are drastically changed as the concentration of MAA increases in PPEA/MAA ratio. Both the Lifshitz−van der Waals and the acid−base components of surface tensions must have played a significant role. The electron-donor (${\gamma }_{M}^{-}$) surface tension components are in majority for 100/0, 75/25 and 50/50 membranes as the magnitude of 63.54, 56.44 and 45.28 mJ/m^2^ respectively, larger than control polyamide, 25/75 and 0/100 membranes. Consequently, the increasing ${\gamma }_{M}^{-}$ of 100/0, 75/25 and 50/50 membranes are resulted in relatively large total interfacial free energy ${\rm{\Delta }}{G}_{MLM}^{TOT}$ in order of magnitude 42.95, 40.37, 26.15 mJ/m^2^ respectively than control polyamide, 25/75 and 0/100 membranes (Table 1). This can be attributed to the high electron donor site at the surface of 100/0, 75/25 and 50/50 membranes which causes change of ${\rm{\Delta }}{G}_{MLM}^{TOT}\,\,$to positive values (hydrophilic surface).

The cause of high interfacial interaction energy with water (hydrophilicity) is a profusion of γ^+^ and γ^−^ surface tension components present at the surface^27^. Among all the prepared membranes, the 100/0, 75/25 and 50/50 membranes have appeared with positive total interfacial free energy ${\rm{\Delta }}{G}_{MLM}^{TOT}$ of 42.95 mJ/m^2^, 40.37 mJ/m^2^ and 26.15 mJ/m^2^. Therefore, it shows appropriate condition to produce more structured water molecule at the interface. The increasingly positive value of ${\rm{\Delta }}{G}_{MLM}^{TOT}$ of 100/0, 75/25 and 50/50 membrane represent a greater hydrophilic repulsion between membrane surface and foulant, and thus lower deposition of foulant is anticipated.

### Organic fouling tendency of prepared FO membranes

In many studies, alginate has been used as model foulant to reveal an organic fouling behavior of the FO membrane^28^. The formation of an alginate layer on FO membrane outcomes in cake-enhanced osmotic pressure, which can result in a decreasing osmotic driving force^20^. The organic fouling of all the membranes are tested in an active layer facing feed solution orientation (AL-FS).

**Figure 6:**
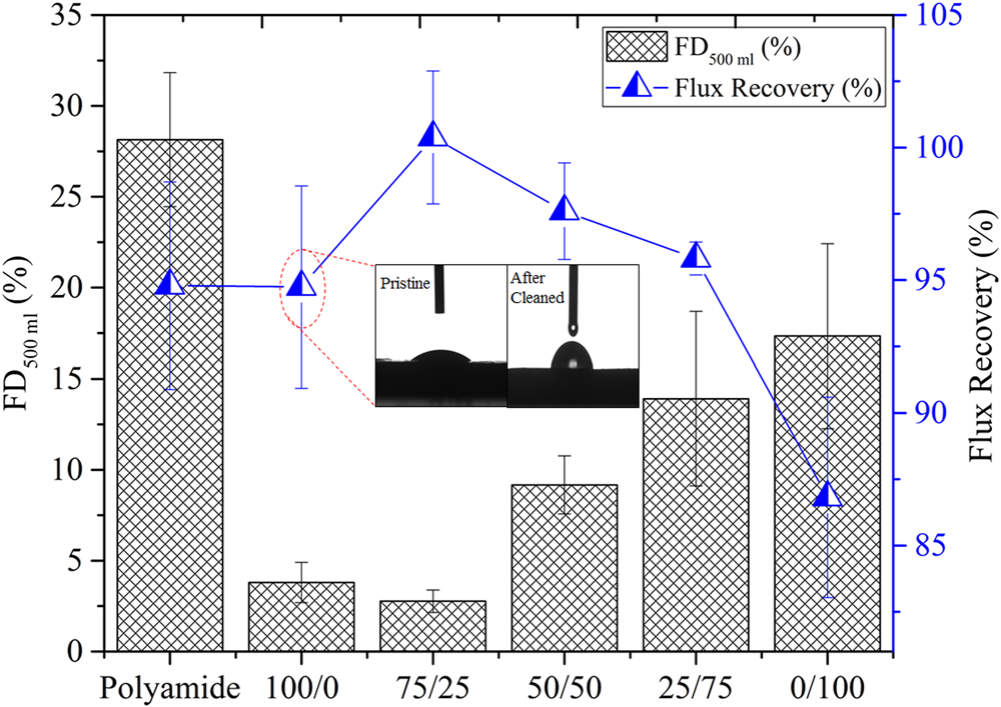
Flux decline FD_500mL_ (%) and flux recovery (%) of alginate fouling experiments of control polyamide and prepared FO membranes.

As shown in Fig. 6, the 100/0, 75/25, 50/50, 25/75 and 0/100 membranes exhibited a flux decline of 3.80 ± 1.11%, 2.77 ± 0.62%, 9.17 ± 1.60%, 13.91 ± 4.80% and 17.35 ± 5.09% respectively, which are significantly less than the control polyamide membrane (28.14 ± 3.70%). In case of 100/0 and 75/25 membranes, a small and insignificant difference in flux decline is observed. The aforementioned quantitative flux decline data of all PPEA/MAA active layer membranes are demonstrated that the photo-polymerized active layer FO membrane has a potential in high fouling resistance than the control polyamide membrane. The small change in flux decline 100/0 and 75/25 (PPEA/MAA) active layer membranes can be attributed to high interfacial free energy (Table 1) which might have caused to a make strong hydrogen bond with water. Consequently, the strong interface layer of water on 100/0 and 75/25 membranes might have acted as a strong barrier between the membrane surface and a foulant. Moreover, the membrane organic fouling tendency is increased as the concentration of MAA increased in the PPEA/MAA ratio.

After the fouling experiment, the membranes are hydro-dynamically cleaned by 15 mM NaCl solution in both feed and draw side of the membrane at a flow rate of 1 L/min. As shown in the Fig. 6, the fouled control polyamide membrane could only be reinstated with 95% water flux. Surprisingly, no significant water flux recovery difference is obtained between the control polyamide and 100/0 fouled membrane (96%), even though there is a very low flux decline of 100/0 membrane. This unexpected result of 100/0 can be hypothetically attributed to the damage of the active layer due to high turbulent flow. In order to verify the damage, the contact angle of the cleaned 100/0 membrane is performed. It is found that the contact angle of the cleaned 100/0 membrane (74 ± 2.1°) is threefold of the pristine 100/0 membrane (24.61 ± 1.2°). The obtained contact angle of cleaned 100/0 membrane revealed that the decreased water flux recovery might be due to the damage of active layer, not because of the membrane fouling. The flux recovery of 75/25, 50/50, 25/75 and 0/100 membranes in a magnitude 100%, 98%, 96% and 87% are recorded. In pressure driven membrane process, a fouling tends to happen in a compact structure, whereas in the FO process, it is always in the form of loosely packed structure. Consequently, in the FO process, it is possible to recover 80–100% flux by only periodic hydraulic rinsing of the membrane. In this concern, the surface properties of FO membrane plays an important role, and the highly energetic and smooth surface (low roughness) of 75/25 membrane can achieve more flux recovery than the control polyamide membrane. The complete data of the baseline, fouling, and recovery experiments are included in Table S1 (Supporting information).

### Intrinsic separation properties of prepared FO membranes

The pure water permeability coefficient *A*_*RO*_, the NaCl permeability coefficient *B*_*RO*_, the NaCl rejection *R*_*s*_, *B*_*RO*_*/A*_*RO*_ ratio and the structural parameter *S* for all the prepared FO membranes are presented in Table 2.

**Table 2:** Intrinsic separation properties of prepared FO membranes.

Membrane	*A*_*RO*_^a^ (LMH bar^−1^)	*R*_*s*_ (%)	*B*_*RO*_^b^ (LMH)	*B*_*RO*_*/A*_*RO*_ (bar)	*S* (µm)
Control Polyamide	15.81 ± 1.55	84.80 ± 3.23	26.57 ± 4.22	1.68 ± 0.36	679
100/0	21.25 ± 1.56	62.22 ± 4.87	122.77 ± 8.64	5.79 ± 0.37	252
75/25	18.62 ± 1.95	80.43 ± 2.21	42.70 ± 2.42	2.29 ± 0.32	338
50/50	17.41 ± 2.07	82.87 ± 1.74	33.98 ± 5.62	1.95 ± 0.30	487
25/75	15.66 ± 1.26	86.43 ± 2.64	22.90 ± 4.60	1.47 ± 0.25	725
0/100	12.92 ± 0.85	87.08 ± 3.65	17.99 ± 3.20	1.40 ± 0.28	826

^a^The pure water permeability (*A*_*RO*_, LMH bar^−1^) is performed in RO mode using DI water as a feed at 10 bar pressure with flat sheet membrane configuration.

^b^The NaCl permeability coefficient (*B*_*RO*_*, LMH*) is conducted in RO mode using 500 ppm NaCl feed solution at 10 bar pressure with flat sheet membrane configuration.

The decreasing pure water permeability (*A*_*RO*_) is observed as the concentration of MAA increases in PPEA/MAA ratio. The control polyamide membrane has shown the pure water permeability (*A*_*RO*_) 15.81 ± 1.55 LMH bar^−1^ significantly less than 100/0 (21.25 ± 1.56 LMH bar^−1^), 75/25 (18.62 ± 1.95 LMH bar^−1^), 50/50 (17.41 ± 2.07 LMH bar^−1^) FO membranes, and slightly more than 25/75 (15.66 ± 1.26 LMH bar^−1^), 0/100 (12.92 ± 0.85 LMH bar^−1^) FO membranes at an applied pressure of 10 bar. A strong trade-off relation is observed in between pure water permeability (*A*_*RO*_) and NaCl rejection (*R*_*s*_), where increasing concentration of MAA in PPEA/MAA ratio is resulted into low water permeability and high NaCl rejection. The 0/100 membrane has revealed maximum *R*_*s*_ (87.08 ± 3.65%) compared with 100/0 (62.22 ± 4.87%), 75/25 (80.43 ± 2.21%), 50/50 (82.87 ± 1.74%), 25/75 (86.43 ± 2.64%) and control polyamide (84.80 ± 3.23%) FO membranes. The above strong trade-off relation between water permeability and NaCl rejection is also a good agreement with the other literature and commercial TFC-FO membrane^29^. These results reveal the formation of denser and lesser permeable active layers at the higher MAA concentrations. The higher pure water permeability of a less dense membrane anticipates an increasing FO water flux due to lower membrane resistance. Furthermore, the lower NaCl rejection of a less dense membrane can be caused for high reverse NaCl flux. The *B*_*RO*_*/A*_*RO*_ ratio of prepared FO membranes is also decreased by the increasing concentration of MAA. In FO applications, the *B*_*RO*_*/A*_*RO*_ ratio is consider as an important parameter to analyse the direct relation of reverse NaCl diffusion. The low *B*_*RO*_*/A*_*RO*_ ratio (i.e. higher selectivity) can be related to the solute rejection enhancement, fouling tendency reduction, and stable FO operation^30^.

According to the high pure water flux (*A*_*RO*_), the prepared membranes may fall under the category of NF like FO membrane. It may also be possible that prepared membranes cannot effectively used for desalination process where NaCl rejection is critical. However, the prepared membrane can have application in wastewater treatment, food processing etc. Because, the Qi *et al*.^31^ has demonstrated the technical feasibility of ultrafiltration (UF) membrane in FO process with poly (sodium 4-styren-sulfonate) (PSS) draw solute and suggested that UF membrane or any porous membrane which has zero rejection for monovalent ion can effectively achieve much higher water flux than RO and NF like membrane when well suited draw solutes are used.

The structural parameter *S* of FO membrane is the property of the substrate which inversely depends on the porosity and, directly depends on the thickness and tortuosity. Despite having the same substrate for FO membrane, the increasing structural parameter *S* is observed as the concentration of MAA increases (Table 2). This can be attributed to the reducing viscosity of PPEA/MAA solution at the increasing concentration of MAA. The high viscosity of 100/0 solution may have been restricted the solution to penetrate deep inside the pores of the substrate. As the quantity of MAA increases, the reduced viscosity of PPEA/MAA solution makes it to penetrate the pores of the substrate. Therefore, the porosity of the substrate is hindered by an active layer formation. Ideally, the *S* value should have been same for all prepared membrane, but practically it is significantly affected by the properties of monomers used in preparation of an active layer.

### Osmotic flux performance of prepared FO membranes

**Figure 7:**
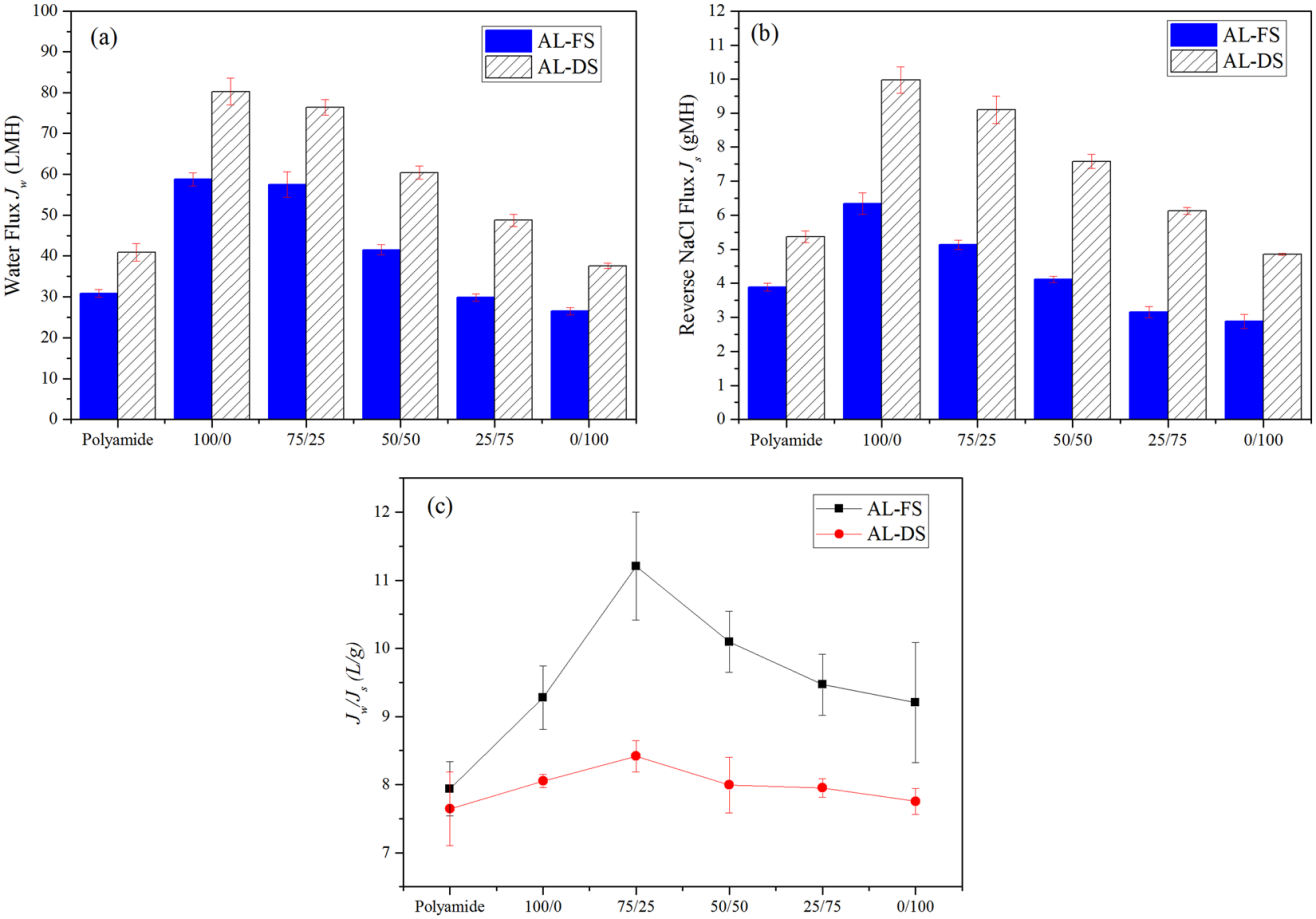
(**a**) Water flux *J*_*w*_ (**b**) Reverse NaCl flux *J*_*s*_, and (**c**) reverse solute flux selectivity *J*_*w*_/*J*_*s*_ of prepared membranes.

Figure 7 shows the osmotic flux performance of prepared FO membranes. The higher water flux (*J*_*w*_) and the higher reverse NaCl flux (*J*_*s*_) of all prepared membranes in AL-DS orientation than AL-FS orientation show the dependency of net osmotic pressure difference on a different degree of dilutive/concentrative internal concentration polarization (ICP)^32^ (Fig. 7). In AL-FS orientation, the control polyamide membrane has exhibited 30.88 ± 0.98 LMH water flux which is significantly lower than 100/0 (58.75 ± 1.62 LMH), 75/25 (57.48 ± 3.02 LMH), and 50/50 (41.52 ± 1.22 LMH) membranes (Fig. 7(a)). However, the 25/75 and 0/100 membranes could produce only 29.85 ± 0.90 LMH and 26.50 ± 0.89 LMH. The decreasing water flux trend is observed as the MAA concentration increases and this can be attributed to the pure water permeability (*A*_*RO*_) reduction of the FO membranes at higher MAA concentrations (Table 2). These changes in water flux may also be attributed to an increasing water contact angle and a decreasing total interfacial free energy ${\rm{\Delta }}{G}_{MLM}^{TOT}$ (Table 1) as the concentration of MAA increases.

The reverse NaCl flux (*J*_*s*_) has been recognized as a second important parameter to evaluate FO membrane performance. From the economic point of view, the reverse diffusion of draw solute is an objectionable phenomenon which also acts as an active origin for fouling and scaling on the active layer of the membrane. As shown in Fig. 7(b), when 10 mM NaCl feed solution and 1 M NaCl draw solution is employed, the *J*_*s*_ of the control polyamide membrane is recorded 3.89 ± 0.12 gMH. Surprisingly, *J*_*s*_ of the 100/0 membrane is increased by 63% (6.33 ± 0.31 gMH) and the reason behind this increasing *J*_*s*_ may be the loosely packed polymeric structure of active layer as its contact with high concentrated draw solution. Moreover, the cause of loosely packed polymeric structure also reflected in *B*_*RO*_*/A*_*RO*_ ratio by showing highest value among all the prepared membranes. As move forward, the the reverse NaCl flux **(***J*_*s*_) of the 75/25 (5.13 ± 0.13 gMH), 50/50 (4.11 ± 0.09 gMH), 25/75 (3.15 ± 0.16 gMH) and 0/100 (2.88 ± 0.20 gMH) membranes reduced in comparison with 100/0 membrane. This can be attributed to the formation of dense and less permeable active layer as the concentration of MAA increases, which constricted the passage for solute ions. These results can also be supported by the increased NaCl rejection (*R*_*s*_) and reduced *B*_*RO*_*/A*_*RO*_ ratio at high concentration of MAA (Table 2). The same performance pattern of water flux (*J*_*w*_) and the reverse NaCl flux (*J*_*s*_) obtained in the case of AL-DS orientation.

In FO membrane synthesis, the structural parameter *S* has always been given more attention due to its direct relation with internal concentration polarization (ICP). Furthermore, it helps to choose specific membrane for particular application^33^. For example, if *S* value of membrane is low then a high pure water permeability (*A*_*RO*_) value (i.e., loosely packed active layer membrane) can play important role in sea water desalination and osmotic power generation where water flux is more important than rejection. If *S* value of the membrane is high then high NaCl permeability coefficient (*B*_*RO*_) or the NaCl rejection (*R*_*s*_) value (i.e., dense active layer membrane) can be considered as a vital parameter in wastewater treatment where rejection of contaminants is more important than water flux. In the case of our prepared FO membranes, 100/0, 75/25 and 50/50 membranes can be used in sea water desalination or osmotic power generation and, 25/75 and 0/100 can be used in wastewater treatment.

On the basis of the aforementioned discussion, it is difficult to find the optimum prepared FO membrane due to the trade-off relation between the water flux and reverse NaCl flux. Hence, we have calculated the reverse solute flux selectivity (RSFS) (*J*_*w*_*/J*_*s*_) for all the prepared membranes because it depends on the selectivity of the active layer and osmotic pressure^34^. Figure 7(c) shows, the reverse solute flux selectivity (RSFS) of all prepared membranes, and among all the prepared membranes, 75/25 is showing highest value of RSFS 11.20 ± 0.79 L/g in AL-FS and 8.41 ± 0.22 L/g in AL-DS.

## Materials and Methods

### Materials

Unless or else stated, all the reagents and chemicals are of analytical grades. For the preparation of substrate membrane, materials and synthesis processes are adopted from our previous work^14^. For the active layer preparation, methacrylic acid (MAA) (SRL Chem, India, M_w_. 86.09), poly(ethylene glycol) phenyl ether acrylate (PPEA) (Sigma-Aldrich, M_w_. 324) as monomer, camphorquinone (CQ) as photo initiator and ethyl 4-dimethylaminobenzoate (EDMAB) as an accelerator are purchased from Sigma-Aldrich. For a control polyamide active layer preparation, trimesoyl chloride (TMC), m-phenylenediamine (MPD), and n-hexane were purchased from Sigma-Aldrich.

### Active layer preparation by photo-polymerization technique

The active layer of FO membranes is prepared by photo-polymerization onto the hand-cast support membranes. The support membranes are fabricated by phase inversion via immersion precipitation of the polyvinyl chloride (PVC) and 2 wt% Layer Double Hydroxide (LDH) composite, pursuing the procedure defined in our previous work^14^. The active layer on the support membrane is then prepared by a photo-polymerization technique^35^, in which poly (ethylene glycol) phenyl ether acrylate (PPEA) and methacrylic acid (MAA) are chosen as monomes in the ratio of (PPEA/MAA) 100/0, 75/25, 50/50, 25/75, 0/100, CQ (0.75 wt%) as a photo-initiator and EDMAB (1 wt%) as an accelerator. Before the photo-polymerization process, the prepared support membranes are covered at a polyester woven fabric side. The prepared mixtures with varying monomer ratio are impregnated on the top surface of the support membrane. Furthermore, all the impregnated supports are polymerized in the presence of visible blue light (λ = 470 nm) at room temperature for 10 min. The photo-polymerized membranes are rinsed with DI water and kept immersed in DI water for 4–5 days. The control polyamide FO membrane is synthesized using interfacial polymerization of MPD and TMC on a hand-cast support membrane. A thorough description of the control FO membrane fabrication is given in previous work^14^.

### ATR-FTIR and TGA/DTG analysis

ATR-FTIR spectra for various functional groups is recorded using Bruker, Germany, 3000 Hyperion Microscope with Vertex 80 FTIR System equipped with ATR unit. TGA for thermal analysis is carried out on a PERKIN ELMER, USA, Diamond TG/DTG instrument, working in N_2_ atmosphere between 30 and 500 °C, with a heating rate of 10 °C/min and N_2_ flow rate of 20 mL/min. The differential thermogravimetric (DTG) plots are calculated as the first derivative of the TGA curve.

### Atomic Force Microscopy (AFM)

The surface morphology and roughness of the membranes are characterized by the AFM instrument. In ambient laboratory conditions at a scan rate of 2 Hz, the 5 µm scans of AFM images are obtained under tapping mode.

### “Wettability” or solid-liquid interfacial free energy

A contact angle experiment is carried out using a sessile drop method with the help of a goniometer OCA 15EC, Dataphysics Instruments, Germany. In order to prepare a sample, the membranes are first vacuum dried in a sealed desiccator. A volume of 1 µL liquid drop is placed on 5 different location of a sample to statistically quantify the error.

A modified form of the Young–Dupré equation is utilized to quantify “wettability” or solid- liquid interfacial free energy as follows^36^,

1 $$-{\rm{\Delta }}{G}_{ML}={\gamma }_{L}(1+\frac{\cos \,\theta }{r})$$

where, ${\gamma }_{L}$ is the total liquid surface tension of DI water (72.8 mJ/m^2^ at 25 °C), $\theta$ is the measured contact angle, *r* is the roughness area ratio, *M* and *L* signified for membrane and liquid respectively. The larger value of $-{\rm{\Delta }}{G}_{ML}\,\,$specify more wettable surface. This equation is accountable for both surface energy and surface morphology, and can give profound information about wettability.

### The polar and apolar surface tension components

The Lifshitz–van der Waals (LW), and acid-base (AB) (electron donor (−), and electron acceptor (+)) contribution for interfacial energy is calculated from the extended Young–Dupré equation^36^.

2 $${\gamma }_{L}(1+\frac{\cos \,\theta }{r})=2(\sqrt{{\gamma }_{M}^{LW}{\gamma }_{L}^{LW}}+\sqrt{{\gamma }_{M}^{+}{\gamma }_{L}^{-}}+\sqrt{{\gamma }_{M}^{-}{\gamma }_{L}^{+}})$$

where, ${\gamma }_{M}^{LW},{\gamma }_{M}^{+}\,\,$and ${\gamma }_{L}^{-}\,\,$are *LW*, electron donor, and electron acceptor components of the membrane surface tension, respectively are determined by measuring contact angles of one apolar (Diiodomethane) and two polar liquids (water and glycerol) of known surface tensions.

Furthermore, the total surface energy of membranes is calculated by the contribution of Lifshitz–van der Waals (LW), and acid-base (AB) components as follow,

3 $${\gamma }_{M}^{TOT}={\gamma }_{M}^{LW}+{\gamma }_{M}^{AB}$$

where,

$${\gamma }_{M}^{AB}=2\sqrt{{\gamma }_{M}^{+}{\gamma }_{M}^{-}}.$$

### “Hydrophilicity” or total interfacial free energy

Van Oss^36^, and Hurwitz *et al*.^37^ suggested the calculation of the total interfacial free energy of membranes from the *LW* and *AB* components of interfacial free energy using following equations;

4 $${\rm{\Delta }}{G}_{MLM}^{TOT}={\rm{\Delta }}{G}_{MLM}^{LW}+{\rm{\Delta }}{G}_{MLM}^{AB}$$

5 $${\rm{\Delta }}{G}_{MLM}^{LW}=2(\sqrt{{\gamma }_{L}^{LW}}-\sqrt{{\gamma }_{M}^{LW}})(\sqrt{{\gamma }_{M}^{LW}}-\sqrt{{\gamma }_{L}^{LW}})$$

6 $${\rm{\Delta }}{G}_{MLM}^{AB}=2\sqrt{{\gamma }_{L}^{+}}(2\sqrt{{\gamma }_{M}^{-}}-\sqrt{{\gamma }_{L}^{-}}\,)+2\sqrt{{\gamma }_{L}^{-}}(2\sqrt{{\gamma }_{M}^{+}}-\sqrt{{\gamma }_{L}^{+}})-2\sqrt{{\gamma }_{M}^{+}{\gamma }_{M}^{-}}-2\sqrt{{\gamma }_{M}^{-}{\gamma }_{M}^{+}}$$

The positive value of ${\rm{\Delta }}{G}_{MLM}^{TOT}$ is considered as non-cohesive when immersed in water or “hydrophilic” and vice versa.

### Organic fouling experiments

In order to analyse the organic fouling of control polyamide and PPEA/MAA photo-polymerized membranes, the experiments are conducted using synthetic wastewater^28^. The composition of synthetic wastewater consists of 250 mg/L sodium alginate (Mw 12–80 kDa, Sigma-Aldrich) dissolved in a solution containing KH_2_PO_4_ (0.45 mM), NaCl (9.2 mM), MgSO_4_ (0.61 mM), NaHCO_3_ (0.5 mM), CaCl_2_ (0.5 mM), and NH_4_Cl (0.93 mM) at pH ~7.4. Each organic fouling experiment is divided into four parts namely baseline, fouling, cleaning and recovery. The NaCl draw solution with a concentration range of 1–4 M was used in every fouling experiment to achieve the constant initial water flux of 21.4 ± 5 LMH. The 5 L volume of a feed and draw solution with a flow rate of 400 mL min^−1^ pumped co-currently into a feed and draw solution channel. The change in draw solution weight with time is monitored by the weighing data logger and each experiment performed until 500 mL permeate is collected (~14 h).

The baseline experiments are conducted with an ionic composition of synthetic wastewater (without sodium alginate) as a feed solution in order to determine permeate flux decline. The baseline experiments ensure that the permeate flux decline is a cause of draw solution dilution and a feed solution concentration in batch mode operation. Whereas, the fouling experiments are carried out by adding 250 mg/L of sodium alginate in an ionic composition of synthetic wastewater. After the fouling experiments, the membranes are cleaned by pumping 15 mM NaCl solution in a feed and draw solution channel at a flow rate of 1 L/min. Finally, the recovery experiments are performed to evaluate the irreversibility of fouling by the same baseline experiment conditions.

For each organic fouling experiment, the flux decline (FD_500mL_, %) was calculated as;

7 $$F{D}_{500ml}=\frac{|{(\frac{{J}_{w,f}}{{J}_{w,o}})}_{baseline}-{(\frac{{J}_{w,f}}{{J}_{w,o}})}_{fouling}|}{{(\frac{{J}_{w,f}}{{J}_{w,o}})}_{baseline}}\,(100)$$

where, *J*_*w,o*_ is initial water flux, and *J*_*w,f*_ is the final water flux after 500 mL permeate has been collected^28^.

### Intrinsic separation properties and structural parameter

The pure water permeability coefficient *A*_*RO*_ (Lm^−2^h^−1^bar^−1^), the NaCl permeability coefficient *B*_*RO*_ (Lm^−2^h^−1^) and the NaCl rejection *R*_*s*_ (%) are determined in reverse osmosis (RO) mode at 10 bar pressure with flat sheet membrane configuration. The effective membrane area in RO module was a 19.6 cm^2^ and salt rejection experiment performed using 500 ppm NaCl feed solution at 10 bar pressure with flat sheet membrane configuration. The following equation is used to calculate the pure water permeability coefficient *A*_*RO*_^14^;

8 $${A}_{RO}=\frac{{\rm{\Delta }}V}{{A}_{m}.{\rm{\Delta }}t.{\rm{\Delta }}P}$$

where *A*_*m*_ is the effective membrane area, and Δ*V* is the permeate volume, Δ*t* is time and Δ*P* is pressure difference.

The NaCl rejection *R*_*s*_ calculated as follow;

9 $${R}_{s}=(1-\frac{{C}_{p}}{{C}_{f}})\times 100 \% $$

where *C*_*f*_ and *C*_*p*_ are the salt concentrations in the feed and permeate solution, respectively. On the basis of the solution–diffusion theory, the NaCl permeability coefficient (*B*_*RO*_) calculated as follow;

10 $$\frac{1-{R}_{s}}{{R}_{s}}=\frac{{B}_{RO}}{{A}_{RO}({\rm{\Delta }}P-{\rm{\Delta }}\pi )}$$

where *A* is water permeability coefficient, Δ*P* is pressure difference and Δ*π* is the osmotic pressure difference across the membrane.

The internal concentration polarization (ICP) model in FO mode is adapted to calculate the structural parameter *S* as follow^14^;

11 $$S=\frac{D}{{J}_{w}}[\mathrm{ln}\,\frac{{A}_{RO}.{\pi }_{draw}+{B}_{RO}}{{A}_{RO}.{\pi }_{feed}+{J}_{w}+{B}_{RO}}]$$

where *D* is the NaCl diffusion coefficient in water;$\,{\pi }_{draw}$ and ${\pi }_{feed}\,\,$is the osmotic pressures of the draw solution and the feed solution, respectively.

### Evaluation of osmotic flux performance

The osmotic flux performance of control polyamide and PPEA/MAA photo-polymerized membranes are tested in the active layer facing feed solution (AL-FS) and active layer facing draw solution (AL-DS) orientation. The flat sheet prepared membranes are sandwiched between two stainless steel plates having an effective area 35 cm^2^ (size of 7 cm × 5 cm). The peristaltic pumps are used to push the feed solution (10 mM NaCl) and draw solution (1 M NaCl). A digital weighing machine attached with AD-1688 weighing data logger - A&D Company Ltd. is used to record weight change in the draw solution tank to calculate water flux (*J*_*w*_). The reverse NaCl flux (*J*_*s*_) is determined by measuring the conductivity of feed solution at the end of each cycle using the conductivity meter (HACH, USA).

The FO water flux (*J*_*w*_) is calculated using below formula;

12 $${J}_{w}=\frac{{\rm{\Delta }}V}{{A}_{m}{\rm{\Delta }}t}$$

where *ΔV* is the volume change of feed solution, *A*_*m*_ is an effective membrane area, *Δt* is the measuring time interval.

The reverse NaCl flux (*J*_*s*_) is determined using formula;

13 $${J}_{s}=\frac{{\rm{\Delta }}({C}_{t}{V}_{t})}{{A}_{m}{\rm{\Delta }}t}$$

where *A*_*m*_ is an effective membrane area, *Δt* is a time interval, *C*_*t*_ and *V*_*t*_ are the salt concentration and the volume of the feed measured at the beginning and the end of the time interval, respectively.

## Conclusion

In this study, the active layer of FO membranes was prepared using the photo-polymerization of poly (ethylene glycol) phenyl ether acrylate (PPEA) and methacrylic acid (MAA). Five different concentration ratio of PPEA/MAA was demonstrated as 100/0, 75/25, 50/50, 25/75 and 0/100. In prepared active layer, the concentration of MAA in PPEA/MAA ratio played a significant inverse role in hydrophilicity, surface energy, organic fouling and osmotic water flux, but surprisingly restrained the reverse NaCl diffusion. Using the surface energy experiment, the organic fouling experiment, and osmotic flux performance, 75/25 FO membrane revealed as a best performed FO membrane. Consequently, this new approach can be an option for fabrication of the future FO membrane. The main intention of this study is to draw attention towards more alternatives for preparation of FO membrane. However, the further investigations are required in a type of monomer used, kinetics and mechanism of photo-polymerization, a study of photo-polymerized active layer-substrate interface, and the optimization of synthesis parameters.

## Electronic supplementary material

Supporting Information

## Data Availability

The data generated during the current study are available from the corresponding author on reasonable request.
