# Supplementary material for: Photo-polymerization as a new approach to fabricate the active layer of forward osmosis membrane
Source: Sci Rep. 2019 Feb 13;9:1937. doi: 10.1038/s41598-018-36346-8 (PMC6374509; doi:10.1038/s41598-018-36346-8)
Supplement: Supplementary file 1 — Supporting Information [file 41598_2018_36346_MOESM1_ESM.docx]

**Photo-polymerization as a new approach to fabricate the active layer of forward osmosis membrane**

**Pankaj M. Pardeshi^†^, Alka A. Mungray ^†^***

^†^Chemical Engineering Department, Sardar Vallabhbhai National Institute of Technology, Ichchhanath, Surat- 395007, Gujarat, INDIA

***Corresponding author:**

Tel.:+91-261-2201716, Email**:** [bag@ched.svnit.ac.in](mailto:bag@ched.svnit.ac.in)

**Supporting Information**

**Table S1.** Organic fouling data of prepared FO membranes

|  | **Initial Flux, J_w,0_ (LMH)** | **Final Flux, J_w,500mL_ (LMH)** | **Flux Decline due to Fouling, FD_500mL_ (%)** | **Flux Recovery after cleaning,**  **J_w,0 (clean)/_J_w,0_ (%)** |
| --- | --- | --- | --- | --- |
| Control Membrane | | | | |
| Baseline | 20.104 | 15.7 | 27.34 | 96.00 |
| Fouling | 22.54 | 12.79 |  |  |
| Recovery | 19.3 | 13.2 |  |  |
| Control Membrane | | | | |
| Baseline | 19.6 | 16.82 | 32.16 | 97.96 |
| Fouling | 23.19 | 13.5 |  |  |
| Recovery | 19.2 | 13.79 |  |  |
| Control Membrane | | | | |
| Baseline | 22.45 | 17.78 | 24.92 | 90.42 |
| Fouling | 22.2 | 13.2 |  |  |
| Recovery | 20.3 | 12.87 |  |  |
| 100/0 Membrane | | | | |
| Baseline | 22.3 | 18.45 | 4.66 | 93.27 |
| Fouling | 20.97 | 16.54 |  |  |
| Recovery | 20.8 | 17.02 |  |  |
| 100/0 Membrane | | | | |
| Baseline | 23.4 | 17.81 | 2.54 | 91.88 |
| Fouling | 22.73 | 16.86 |  |  |
| Recovery | 21.5 | 17.22 |  |  |
| 100/0 Membrane | | | | |
| Baseline | 21.5 | 19.2 | 4.17 | 99.07 |
| Fouling | 20.86 | 17.85 |  |  |
| Recovery | 21.3 | 16.88 |  |  |
| 75/25 Membrane | | | | |
| Baseline | 21.23 | 18.88 | 3.47 | 98.44 |
| Fouling | 20.2 | 17.34 |  |  |
| Recovery | 20.9 | 16.83 |  |  |
| 75/25 Membrane | | | | |
| Baseline | 21.41 | 18.73 | 2.30 | 99.48 |
| Fouling | 20.3 | 17.35 |  |  |
| Recovery | 21.3 | 17.22 |  |  |
| 75/25 Membrane | | | | |
| Baseline | 20.83 | 18.55 | 2.52 | 103.21 |
| Fouling | 19.93 | 17.3 |  |  |
| Recovery | 21.5 | 16.87 |  |  |
| 50/50 Membrane | | | | |
| Baseline | 19.43 | 17.01 | 8.9 | 97.27 |
| Fouling | 20.85 | 16.63 |  |  |
| Recovery | 18.9 | 16.31 |  |  |
| 50/50 Membrane | | | | |
| Baseline | 23.76 | 17.88 | 7.72 | 95.95 |
| Fouling | 24.51 | 17.02 |  |  |
| Recovery | 22.8 | 17.66 |  |  |
| 50/50 Membrane | | | | |
| Baseline | 25.01 | 19.42 | 10.9 | 99.56 |
| Fouling | 22.65 | 15.67 |  |  |
| Recovery | 24.9 | 16.77 |  |  |
| 25/75 Membrane | | | | |
| Baseline | 20.13 | 15.6 | 10.32 | 96.37 |
| Fouling | 19.34 | 13.44 |  |  |
| Recovery | 19.4 | 14.05 |  |  |
| 25/75 Membrane | | | | |
| Baseline | 18.87 | 16.34 | 19.36 | 95.91 |
| Fouling | 22.67 | 15.83 |  |  |
| Recovery | 18.1 | 12.55 |  |  |
| 25/75 Membrane | | | | |
| Baseline | 20.81 | 16.01 | 12.05 | 95.14 |
| Fouling | 21.83 | 14.77 |  |  |
| Recovery | 19.8 | 13.85 |  |  |
| 0/100 Membrane | | | | |
| Baseline | 23.76 | 15.85 | 17.61 | 85.43 |
| Fouling | 21.47 | 11.8 |  |  |
| Recovery | 20.3 | 12.55 |  |  |
| 0/100 Membrane | | | | |
| Baseline | 20.77 | 12.98 | 22.3 | 83.91 |
| Fouling | 23.6 | 11.46 |  |  |
| Recovery | 17.43 | 11.77 |  |  |
| 0/100 Membrane | | | | |
| Baseline | 24.7 | 16.32 | 12.13 | 91.09 |
| Fouling | 22.41 | 13.01 |  |  |
| Recovery | 22.5 | 12.8 |  |  |

**Fig S1**. The plausible mechanism of photo-polymerization of PPEA.

In Fig. S1, the free radical formation step, CQ and EDMAB initiator were activated with the use of blue light and resulted in a highly reactive CQ and EDMAB free radicals. In initiation step, resultant free radical break the acrylate C=C bond of PPEA monomer, and a free radical moved to the adjacent carbon atom. The reaction propagated by attaching a methylene group of a next PPEA monomer to the free radical carbon atom and a reaction continue till complete polymerization and a termination.^20^

**Fig S2.** The plausible mechanism of photo-polymerization of mixture PPEA/MAA in the ratio of 75/25, 50/50 and 25/75.

In Fig S2, at initiation step, the formed free radical reacted with the methylene group of MAA/PPEA monomer and attached to one side of double bond creating active carbon. In chain propagation step, the active carbon of MAA/PPEA monomer attached in the same manner to new monomer molecules and backbone developed through repeated attachment of active end to new monomer until termination of the reaction.

**Fig S3.** The plausible mechanism of photo-polymerization of MAA.

Similarly, on the basis of FTIR spectra of MAA and Poly (MAA) (0/100), the plausible mechanism of photo-polymerization of MAA was predicted as given in Fig. S3.
